# Paradoxical Roles of Desmosomal Components in Head and Neck Cancer

**DOI:** 10.3390/biom11060914

**Published:** 2021-06-20

**Authors:** Yin-Qiao Liu, Hai-Ying Zou, Jian-Jun Xie, Wang-Kai Fang

**Affiliations:** 1 Department of Biochemistry and Molecular Biology, Shantou University Medical College, Shantou 515041, China; 17yqliu1@stu.edu.cn (Y.-Q.L.); zouhy@stu.edu.cn (H.-Y.Z.); 2 Precision Medicine Research Center, Shantou University Medical College, Shantou 515041, China

**Keywords:** desmosomes, head and neck cancer, desmosomal cadherins, armadillo proteins, plakin proteins

## Abstract

Desmosomes are intercellular adhesion complexes involved in various aspects of epithelial pathophysiology, including tissue homeostasis, morphogenesis, and disease development. Recent studies have reported that the abnormal expression of various desmosomal components correlates with tumor progression and poor survival. In addition, desmosomes have been shown to act as a signaling platform to regulate the proliferation, invasion, migration, morphogenesis, and apoptosis of cancer cells. The occurrence and progression of head and neck cancer (HNC) is accompanied by abnormal expression of desmosomal components and loss of desmosome structure. However, the role of desmosomal components in the progression of HNC remains controversial. This review aims to provide an overview of recent developments showing the paradoxical roles of desmosomal components in tumor suppression and promotion. It offers valuable insights for HNC diagnosis and therapeutics development.

## 1. Introduction

Desmosomes are membrane structures that mediate cell-cell junction and adhesion. This complex connects to the cytoplasmic intermediate filaments (IF) through desmosomal components interactions and acts as a resistance to mechanical stress in tissues [1]. Desmosomes are most abundant in the heart and skin tissues, whose dysfunction induces various desmosome-related diseases, such as skin disease, heart disease, and cancers [2].

The core components of desmosomes are three protein subfamilies: desmosomal cadherins, armadillo proteins, and plakin proteins [3]. Desmosomal cadherins are a class of transmembrane proteins composed of desmoglein (DSG) and desmocollin (DSC), which not only mediate cell adhesion and desmosome assembly but also act as signaling scaffolds for cell movement [4]. The armadillo family consists of plakophilins (PKP) and plakoglobin (PG), which binds to an intracellular fragment of desmosomal cadherins. The plakin family is composed of desmoplakin (DSP), which is connected to IF through the C-terminal domain [5]. Another essential component is the p53 apoptosis effecter, which is related to the PMP-22 (PERP). Although PERP has been shown to play a critical role in desmosome assembly and maintenance, its interaction partners are currently unknown [6,7]. The group of these specific desmosomal components associated with the cytoskeleton is essential for maintaining tissue integrity and architecture.

Tumor progression is often accompanied by the loss of cell-cell adhesion [8,9]. Moreover, the desmosomal component reduction is associated with tumor development [10,11,12,13,14]. The decrease of DSG1, DSC2, DSC3, DSG3, PG, PKP1-3, and DSP expression associated with poor prognosis in patients with multiple cancers such as head and neck cancer, colon cancer, skin cancer, esophageal cancer, lung cancer, cervical cancer, and gastric cancer [15,16,17,18,19,20,21,22]. Confusingly, upregulation of several desmosomal components, including DSG2, DSG3, PKP3, and PKP1, was observed in the development of various human cancers, including skin cancer, lung cancer, head and neck cancer, prostate cancer, colon cancer, cervical cancer, breast cancer, and esophagus cancer, often correlating with accelerated proliferation, increased metastasis or poor prognosis of tumors [23,24,25,26,27,28,29,30,31,32]. The paradoxical roles of desmosome family members in tumor progression suggest that their underlying mechanisms in cancer are two-sided and intricate. In addition to changes in desmosome molecular expression, subcellular location [29], interacting proteins [33], and post-translational modification (PTM) [34] may also affect the role of desmosome in cancer, indicating that their function may depend on the specific tumor microenvironment.

Head and neck cancer (HNC) is a set of cancers in the upper aerodigestive tract, including the oral cavity, nasopharynx, oropharynx, hypopharynx, and larynx [35]. Most head and neck cancers are squamous cell carcinomas (SCC). The most important risk factors of HNC are tobacco and alcohol. However, increasing evidence has confirmed human papillomavirus (HPV) is a causal factor for HNC [36,37]. Additionally, the risk of HNC for individuals with cancer susceptibility syndromes [38], body mass index (BMI) [39], and occupational factors [40] have been also assessed. HNC is the eighth most common type of cancer in the world. [41,42]. According to Global Cancer Statistics 2020 [43], the incidence of HNC is about 870,000 cases, accounting for 4.5% of all malignant cancers. Further elucidating the molecular events involved in HNC development may help identify potentially effective biomarkers and provide new procedures for targeted therapy. The pathogenesis of HNC is a multistep process involving the progressive accumulation of molecular alterations. Altered expression/localization of desmosome family members also plays a vital role in the development of HNC. In this paper, we summarize the expression and function of desmosomal components in HNC. In addition, we also discuss the contradictory roles of desmosomal components in HNC and put forward our views and prospects.

## 2. The Loss of Desmosome during HNC Development

HNC development involves multiple histopathological steps and is accompanied by significant morphological changes in the epithelial tissue. These changes in histopathological features are one of the critical bases for the clinical diagnosis of HNC. In normal squamous cells, desmosomes are tightly arranged between the cells. During oral tumorigenesis, the number of desmosomes in altered premalignant epithelial cells reduces, resulting in loose cell-to-cell adhesion. As the tumor progresses, the number of desmosomes in the infiltrating carcinoma decreases significantly, and cell adhesion is lost [44,45,46]. A similar situation exists in the in vitro co-culture 3D model to simulate the tongue tumor [47].

**Figure 1:**
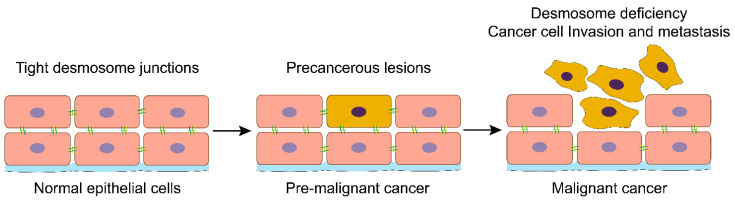
Loss of desmosomes in the progression of head and neck cancer. Schemes follow the same formatting. Normal head and neck epithelial cells have tight desmosome junctions to ensure that the epithelial tissue maintains the correct cell-cell spatial conformation. Activation of oncogenes or suppressing cancer suppressor genes will drive the transformation of normal epithelial cells into cancer cells. With the malignant progression of head and neck cancer, the desmosomes and other cell-cell adhesions are lost, and cancer cells lose their in-situ bondage, thus acquiring the ability of invasion and metastasis.

Moreover, during malignant transformation of oral mucosal epithelium, the number of desmosomes reduced in animal models treated with the chemical carcinogen 9.10-dimethyl-1.2-benzoanthracene (DMBA) [48,49]. Consistent with these conclusions, Kellokumpu et al. found an increase in desmosome abundance in tissue samples from HNC patients treated with radiotherapy [50,51]. In addition, there is a significant correlation between desmosome loss and tumor metastasis [52,53,54] (Figure 1).

To determine the contribution of desmosome dysfunction to cancer development, Beaudry et al. constructed a chronic UVB-induced SCC tumor model in PERP-conditioned knockout mice [55]. Loss of PERP leads to both compromised desmosome-mediated intercellular adhesion and reduced desmosomal number. Interestingly, although PERP-deficient tumors showed distinct downregulation of desmosomal components, adhesion junction components were maintained [55]. This study suggests that the loss of PERP and desmosomes promotes cancer through specific mechanisms rather than general changes in differentiation status. Desmosome loss occurs before the loss of adhesion connections, and desmosome loss drives early tumor invasion before the downregulation of adhesion connections [56]. In oral squamous cell carcinoma (OSCC), loss of PERP is related to increased tumor aggressiveness and worse local control. In addition, the expression of PERP is downregulated in most invasive SCC but not in actinic keratosis, indicating that loss of PERP is an early event in oral carcinogenesis [57]. Loss of PERP expression in nonneoplastic epithelial cells adjacent to the surgical margin in patients with head and neck squamous cell carcinoma (HNSCC) is associated with a higher risk of local recurrence [58].

Alterations in desmosome localization cause the loss of cell-cell adhesion. Desmosome-related molecules detach from the membrane and accumulate in the cytoplasm, resulting in a marked enlargement of the intercellular space [56,59]. Previous studies proved that desmosome internalization into the cytoplasm contributes to the loss of intercellular contract and disease progression [60]. The alterations of desmosome expression and localization are vital manifestations of oral tumor development, which can be used as a molecular indicator for early diagnosis and treatment. For example, local administration of heparin-binding epidermal growth factor (HB-EGF), an effective stimulator for preventing radiation-induced oral mucositis, increased the quality and quantity of desmosome in the tongue and buccal mucosa of mice [61]. *N*,*N*-dimethylformamide (DMF) is an anti-tumor compound that can induce cancer cells to form better-differentiated phenotypes. Nude mice with head and neck xenografts treated with DMF show higher cell differentiation and increased desmosomes [62].

Taken together, strict regulation of desmosome expression and assembly is necessary for normal tissue homeostasis. Due to the vital role of desmosome in maintaining the stability of head and neck tissues, the variation of desmosomal components may play an essential role in the progression of HNC.

## 3. The Expression and Significance of Desmosomal Components in HNC

The expression and clinical significance of desmosomal components are different in head and neck cancer (Table 1). The levels of DSC1 [63] and PKP2 [64] are significantly higher in HNC tissues than para-tumor tissues, and their expression predicts a poor outcome. These studies indicate that DSC1 and PKP2 play a potential oncogenic role in the development of HNC. Conversely, the levels of DSC2 [65], DSC3 [66,67], DSG1 [15,68], PKP3 [64,69,70], and DSP [71,72] are significantly lower in HNC tissues and their expression indicate a good clinical outcome. The results suggest that these desmosomal components may work as tumor suppressors in HNC.

However, the functional study of gain or loss and immunohistochemistry identification of desmosomal components have revealed contradictory roles for some desmosome members, including DSG2 [65,73,74,75], DSG3 [26,65,66,76], PKP1 [27,77,78], and PG [70,79,83] in HNC, suggesting that the role of the desmosomal components in HNC may be affected by some other signal transduction molecules or/and modifications related to the tumor microenvironment. Additionally, like other tumors, subcellular location, PTM, inactivation by proteolytic cleavage, and the biogenesis of regulating extracellular vesicles (EVs) may also affect the role of desmosomes in HNC. In the following sections, each desmosomal component will be discussed in detail (Table 2), aiming to understand their significance in HNC.

## 4. The roles of Desmosome in HNC

### 4.1. Desmosomal Cadherins

**Figure 2:**
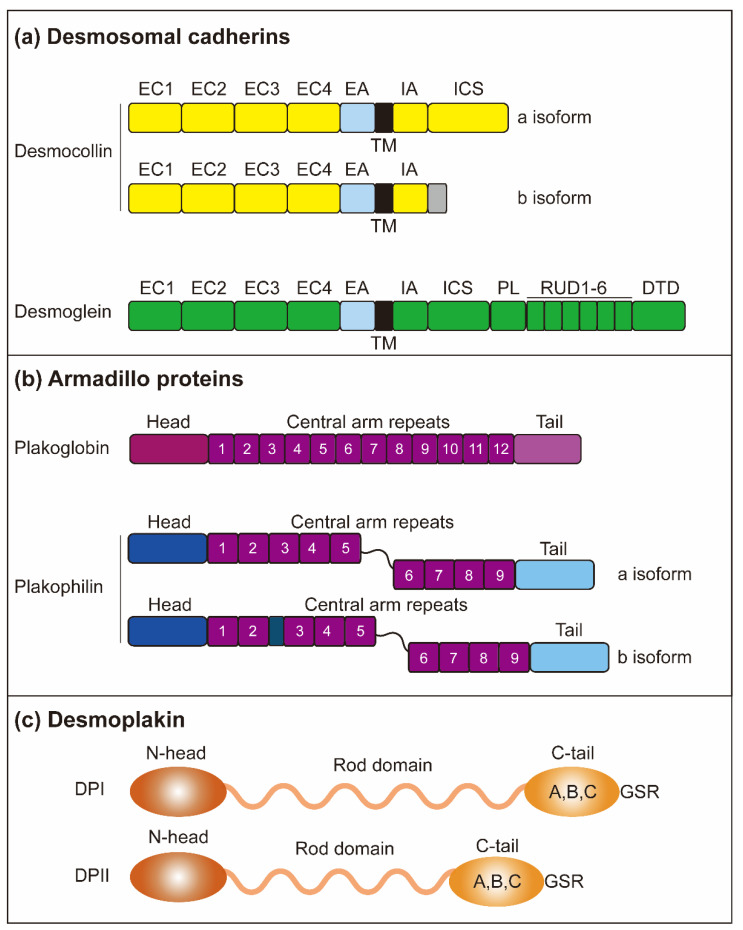
Structure of desmosomes. (**a**) The desmosomes are mainly composed of three protein subfamilies: desmosomal cadherins, armadillo proteins, and plakin proteins. Desmosomal cadherins contain DSGs (DSG1-4) and DSCs (DSC1-3) and are involved in crossing the plasma membrane. Both DSG and DSC contain four highly conserved EC domains, followed by EA. Both DSG and DSC contain a single transmembrane domain (TM). Intracellular, DSGs and DSCs include IA and ICS. The DSGs contain an additional PL domain and five duplicate RUD domains. DSC consists of two isoforms, DSC-a and DSC-b, which have different intracellular segments. DSC-a and DSC-b isoforms contain the IA domain, while DSC-b has a unique cytoplasmic sequence. (**b**) Armadillo proteins contain PG and PKP (PKP1-3). PG and PKP both have a short amino-terminal (head) domain and carboxyl-terminal (tail) domain. PG contains 12 arm repeats in the middle of the protein, while PKPs have nine-arm repeats. The PKP arm domain is interrupted between repeats 5 and 6 by a sequence, introducing a kink throughout the structure. (**c**) The DP domain indicates that N-head mediates the interaction of armadillo family proteins, and the C-tail mediates the interaction of intermediate filaments through GSR.

In humans, desmosomal cadherins consist of DSG1-4 and DSC1-3. These proteins include five extracellular domains (ECs) that allow desmosomes to exhibit calcium-dependent assembly and adhesion [104]. Following the transmembrane domain, the cytoplasmic side contains an intracellular anchor (IA) domain and an intracellular cadherin-typical sequence (ICS) domain. Interestingly, the DSGs have additional sequences, including a proline-rich linker (PL) region, a repeat unit domain (RUD), and a DSG terminal domain [105]. The DSC gene is alternatively spliced to produce an “a” and a “b” isoform [106] (Figure 2). DSG2 and DSC2 are widely expressed in various tissues, while other desmosomal cadherins are mainly present in the stratified epithelium, and their distribution and expression are related to differentiation and are tissue-specific.

**Figure 3:**
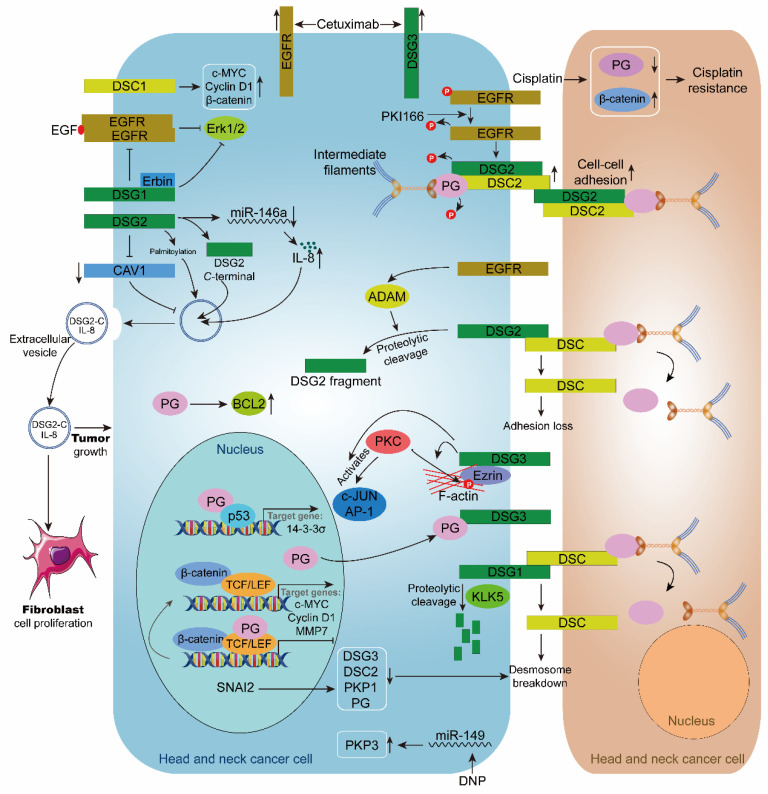
Role of desmosome in head and neck cancer. The inhibitor PKI166 inhibits the phosphorylation of EGFR, DSG2, and PG, and upregulates the expression of DSG2 and DSC2, thereby stabilizing cell-cell adhesion. Cisplatin upregulates β-catenin and down-regulated PG, leading to cisplatin resistance in head and neck cancer cells. Cetuximab upregulates the expression of EGFR and DSG3 in head and neck cancer cells. EGFR enhances ADAM-mediated intracellular hydrolysis of DSG2, resulting in desmosome loss. DSG3 enhances PKC-mediated phosphorylation of c-JUN, AP-1, and Ezrin. KLK5 cleaves DSG1, causing desmosome breakdown. Transcription factor SNAI2 causes desmosome breakdown by downregulating DSG3, DSC2, PKP1, and PG. PG inhibits the transcriptional regulation of TCF/LEF in the nucleus. When PG is recruited to the cell membrane by DSG3, TCF/LEF transcription is activated, and the expressions of target genes c-MYC, Cyclin D1, and MMP7 are upregulated. PG interacts with p53 to promote the expression of the target gene 14-3-3σ. DSG1 and Erbin inhibit the EGF/EGFR/Erk1/2 signaling pathway. DSG2 promotes extracellular vesicle secretion by inhibiting CAV1. DSG2 palmitoylation stimulates extracellular vesicle secretion. DSG2 C-terminal and IL8 are present in extracellular vesicles and encourage the growth of fibroblasts and HNC cells. DSC1 upregulates the expression of c-MYC, Cyclin D1, and β-catenin. DNP promotes PKP3 expression by inhibiting miR-149. PG can upregulate BCL2.

Recently, some experiments have shown that desmosomal cadherin has a tumor-suppressing effect in HNC. In contrast, others have provided evidence of oncogenic function, which may reflect context-dependent differences in their role in HNC. It suggests that the part of desmosomal cadherins in HNC is complicated and contradictory. Next, we will discuss the function of desmosomal cadherins in HNC from these two aspects (Figure 3).

#### 4.1.1. Desmosomal Cadherins Act as a Suppressor in HNC

Many studies have revealed that upregulation of desmosomal cadherin protein enhances cell adhesion and inhibits HNC progression. For example, DSC3 protein and mRNA are upregulated in TP53-mutated maxillary carcinoma accompanied by a marked increase in membrane localization, indicating enhanced cell adhesion. TP53 mutated tumors have phenotypes that are the opposite of cancer progression and malignant transformation [84]. In addition, OSCC cells exhibited DSG1 cleavage, which was related to the loss of cell-cell adhesion function. Protease inhibitor treatment and siRNA silencing of serine proteinase kallikrein 5 (KLK5) expression blocked the hydrolysis process of DSG1, thereby enhancing cell adhesion [85]. Except for the adhesion function, DSG1, through its interaction with ErbB2 Interacting Protein (Erbin), downregulates invadopodia signaling by dampening Epidermal Growth Factor Receptor (EGFR)/Erk activation, which ultimately leads to a decrease in invadopodia formation and matrix degradation [86]. These reports suggest that the expression of DSC3 and DSG1 has an inhibitory effect on HNC.

The low expression of DSG2 in HNC is in line with the result of its functional study, in which DSG2 may act as a tumor suppressor to enhance intercellular adhesion. In support, after treating OSCC cells with the proteasome inhibitor borosomide, the level of DSG2 is reduced, and the cell-cell mechanical adhesion is decreased [89]. In addition, OSCC cells treated with EGFR small molecule inhibitor PKI166 and monoclonal antibody C225 (cetuximab) were found to have accumulated levels of DSG2 and DSC2, which were recruited to cell-cell borders. In this case, inhibition of EGFR downregulates matrix metalloprotease (MMP)-dependent extracellular domain shedding of DSG2. Furthermore, these morphological and molecular changes are accompanied by an increase in cell-cell adhesion [90]. Recent studies have further demonstrated that EGFR and MMP inhibition enhances DSG2 on the cell membrane surface by interfering with its accumulation in the internalized cytoplasmic pool [91]. Moreover, the silencing of multiple ADAM (a disintegrin and metalloprotease) family members also prevented the internalization of DSG2. These reports suggested that EGFR and ADAMs synergistically regulate the cleavage and endocytic trafficking of DSG2 [90,91]. In addition to enhancing cell adhesion, in anaplastic thyroid cancer (ATC) knockdown of DSG2 enhanced cell invasion and migration by activating the hepatocyte growth factor receptor (HGFR, c-Met)/Src/Rac1 signaling axis [73].

In OSCC, there is a potential link between DSG3 and EGFR, and the expression of DSG3 was significantly increased after treatment with cetuximab, an inhibitor of EGFR. Furthermore, high calcium-associated DSG3 induction enhanced the efficacy of cetuximab in cetuximab-low-sensitive cell lines by up to 23% [92]. Differential expression analysis in oral leukoplakia (OL) tissues and OSCC tissues showed mild to severe loss of the DSG3/γ-catenin complex and a transition from membranous to cytoplasmic expression, resulting in perinuclear aggregation, which was directly related to the grade of dysplasia [95].

#### 4.1.2. Desmosome Cadherins Act as an Oncogene in HNC

In contrast, a low expression of DSC1 reduced the proliferation and invasion of HNC cells accompanied by decreased levels of β-catenin, c-myc, and cyclin D1 proteins [63]. In addition, DSG2 was also involved in the malignant phenotype of HNC as an oncogenic gene. We mentioned that DSG2 is highly expressed in HNC, so how does it function? Overexpression of DSG2 leads to the release of EVs and promotes the progression of tumors. First, DSG2-EVs activate mitogenic pathways such as ERK1/2 and Akt signaling pathways and enhance fibroblast cell proliferation [88]. In this report, C-terminal fragments of DSG2 and EGFR were enriched in serum-derived EVs from patients with HNSCC.

Moreover, DSG2 regulates the biogenesis of EVs by controlling the shedding of extracellular domains through MMP and caveolin-1 (CAV1) [88]. Second, in SCC, upregulated DSG2 promotes tumor growth by down-regulating miR-146a, resulting in increased expression of Interleukin 8 (IL-8) and release in EVs. In vivo, associations between DSG2 and IL-8 have been demonstrated in patients with HNSCC. Furthermore, the oncogenic ability of DSG2 in SCC was correlated with the EV level [87]. These results suggest that intercellular communication can be coordinated through the secretion of DSG2 and EVs, critical for tumor growth, and may serve as a potential biomarker to guide treatment regimens.

Similarly, in addition to enhancing cell adhesion, DSG3 also promotes cancer progression through intercellular signaling molecular transduction. How does DSG3 play an oncogenic role in HNC? Firstly, Brown et al. showed that DSG3 promoted invasion and migration of OSCC cells. Mechanisms demonstrated that DSG3 regulates c-Jun/activator protein 1 (AP-1) activity and protein kinase C (PKC)-mediated phosphorylation of Ezrin-Thr567, which contributes to the motility of cancer cells [93]. Secondly, DSG3 may facilitate the proliferation of HNC cells by recruiting PG and subsequently activating the expression of TCF/LEF downstream target genes c-myc, cyclin D1, and MMP-7 [94]. Finally, buccal squamous cell carcinoma cells overexpressing full-length or C-terminated (Δ238 and Δ560) mutants of DSG3 migrated more rapidly than empty vector control cells [107].

#### 4.1.3. PTM and Subcellular Locations of Desmosomal Cadherins

PTM and subcellular location may profoundly affect the role of desmosomal cadherins in HNC. The localization of DSC2, DSC3, and DSG1 on the membrane was significantly reduced in oral cancer and was internalized into the cytoplasm. However, this significant difference in localization with normal tissue samples suggests a decrease in desmosome-mediated intercellular adhesion during the progression of HNC [65]. In addition to the subcellular location of desmosomal cadherins, post-translational modification can also affect cell-cell adhesion—the assembly of desmosomes following EGFR inhibition associates with decreased tyrosine phosphorylation of both DSG2 and PG. Slightly surprisingly, phosphorylation of the adherens junction components E-cadherin and β-catenin did not change after PKI166 treatment. These desmosome-specific alterations in tyrosine phosphorylation were accompanied by recruitment of DSP cell-cell borders and tethering of keratin IF to the plasma membrane [90]. In support, IF-desmosome attachment strengthens cell-cell adhesion [108]. In addition, DSG2 regulates the biogenesis of EVs in a palmitation-dependent manner. The palmitoylation of DSG2 alters the trafficking of membrane raft proteins and early endosomal proteins. In the xenograft model, DSG2 promoted tumor growth and reduced this effect considerably with overexpression of non-palmitoylated DSG2 in cells [87]. These phenomena suggest that the post-translational modification of desmosomal cadherins is crucial in deciding its role in HNC.

#### 4.1.4. Diagnostic and Therapeutic Potential of DSG3 in HNC

Cancer-related deaths are mainly due to metastases, not the primary tumor [109]. HNSCC is one of the most common metastatic cancers [110]. Although some advances have been achieved in diagnosis and treatment, the five-year survival rate remains low [111]. It is principally attributed to the lack of essential biomarkers for diagnosis, prognosis, and detection of tumor response to therapy. DSG3 is upregulated in HNSCC [26,76,111,112], and its potential as a diagnostic and prognostic marker has been studied.

Metastasis to regional lymph nodes is common in HNSCC due to a rich lymphatic network and many lymph nodes in the neck region [113,114]. Moreover, the diagnosis of cervical lymph node metastasis is a necessary condition for clinical staging and treatment, which is also an essential factor affecting the prognosis of HNSCC [36,115,116,117]. However, the accurate diagnosis of lymph node metastasis still has limitations. Patients with clinically negative lymph nodes tend to have a higher recurrence rate [118,119]. Sentinel lymph node biopsy is a feasible measure to identify patients with negative nodes [120]. In contrast, its potential is limited by the lack of accurate methods and markers to detect metastatic nodes. DSG3 has been identified as a biomarker for precise detection of HNC lymph node metastasis and can clearly distinguish clinically positive and negative lymph nodes [121,122,123,124]. In addition, low concentrations of DSG3 can be detected as a reliable biomarker for HNSCC lymph node metastasis using a 3D-printed microfluidic immunoassay [121,125]. These advances suggest that DSG3 can help clinicians identify false-negative lymph node metastasis to improve diagnostic accuracy and provide treatment strategies for patients with HNC.

DSG3 protein was differentially overexpressed in HNC cells with 11q13 amplification. Moreover, the piggyback assays demonstrated that the expression of DSG3 is sufficient to induce antibody internalization and cell killing in highly expressing cell lines [126]. In addition, RNAi-mediated DSG3 silencing reduced xenograft tumor growth and metastasis in HNC cell lines [26]. It suggests that DSG3 has potential as a drug target for HNC, offering new advances in inpatient therapy.

### 4.2. Plakophilins

Armadillo proteins include the plakophilins PKP1, 2, and 3. PKP4 is also often associated with this family, but its presence in desmosomes is still controversial [127,128]. PKP1 and two each exist as two isoforms, a short “a” form, and a longer “b” form. The difference is that the longer isoform adds several amino acids in the arm-repeat domain: PKP1 adds 21 amino acids in the third, and PKP2 adds 44 amino acids in the fourth [129]. Moreover, the identified PKP binding partners are related to the PKP amino-terminal head domain, while the precise role of the PKP central arm repeating domain remains unknown [130] (Figure 2). Similar to desmosomal cadherins, PKP1-3 shows a specific expression pattern in tissues and differentiation [131]. PKP1 is highly expressed mainly in the suprabasal layers of stratified epithelia, whereas PKP2 is widely expressed in epithelial and non-epithelial tissues such as myocardium and lymph nodes [132,133]. PKP3 is present in simply stratified epithelial cells [134]. In addition, PKP1 and PKP2 are localized in the nucleus, but how these expression patterns and their localization are combined with the potential functions regulated by various PKP isoforms is unknown. 

#### 4.2.1. PKP1 and PKP3 Act as a Suppressor in HNC

Abnormal expression and localization of PKP have been related to various diseases and cancers. The reduction of PKP1 expression is correlated with aggressive characteristics in HNC. Furthermore, PKP1 was prominently distributed in the cytoplasm of tumors with local recurrence regardless of the presence of membrane immunoreactivity [64]. The loss of PKP3 in both nasopharyngeal carcinoma (NPC) and OSCC were associated with tumor progression and metastasis [64,69,70]. In addition, PKP3 is more frequently localized in the cytoplasm of oral cancer tissue than PKP1 [64]. Therefore, PKP1 and PKP3 play a tumor suppressor role in HNC.

With the development of HNC, desmosome assembly is often disrupted and lost. PKP plays a crucial role in desmosome stabilization and is also one of the critical proteins involved in tumor development. Therefore, it is necessary to further understand the mechanisms by which PKP1 and PKP3 are downregulated and how their loss promotes cancer progression (Figure 3). In OSCC, the reduced expression of PKP1 caused a marked redistribution of DSP from the cell borders to diffuse cytoplasmic localization, resulting in decreased desmosome assembly and altered cell-cell adhesion, thereby increasing tumor cell motility and invasion [96]. The Snail family of zinc-finger transcription factors, including slugs, has been shown to play an essential role in epithelial-mesenchymal transformation (EMT) in various tissues, and slug expression is associated with increased metastatic behavior of tumor cells, which is similar to the EMT phenotype [135]. A decrease of DSG3, DSC2, and PKP1 was observed in slug-expressed cells, inducing EMT characterized by desmosome loss of adhesion. Furthermore, detection of the PKP1 promoter region revealed a putative E-box sequence that may act as a slug-binding element, but the function of this site needs further investigation [97]. *N*,*N*′-dinitrosopiperazine (DNP), an NPC-specific carcinogen, can inhibit the expression of PKP3 by upregulating miR-149, increase the migration, invasion, and adhesion of cells, and finally promote NPC metastasis [98]. In conclusion, the mechanism of PKP1 and PKP3 provides new insights into the metastatic research of HNC and new therapeutic strategies.

#### 4.2.2. PKP2 Acts as an Oncogene in HNC

Interestingly, immunohistochemical results showed that PKP2 expression was more robust in metastatic tumors than in non-metastatic tumors [64], suggesting that PKP2 plays a potential oncogenic role in the development of oral cancer. However, further studies are needed to determine how PKP2 is involved in oral cancer metastasis.

### 4.3. Plakoglobin

The other armadillo member in the desmosome is PG, a homolog of β-catenin, also known as junction plakoglobin (JUP), and gamma catenin (γ-catenin). Structurally, the protein contains 12 repeat arms with different amino and carboxy-terminal domains on both sides [136]. Deletion mutation studies indicate that several repeating arms near the amino and carboxyl terminus of the protein are the key to the binding of desmosomal cadherin [137,138]. The central armadillo domain of PG interacts with DSP, which binds intermediate filaments to desmosome plaques [139,140] (Figure 2). In addition, PG is located in desmosome and adhesion junctions, but its affinity with desmosomal cadherins is much higher than that of E-cadherin [137]. Similar to PKP, PG also exists in cytoplasm and the nucleus, and it has some known nuclear functions, such as transcriptional regulation and inhibition of Wnt/β-catenin signaling [11,141], suggesting that in addition to cell-cell adhesion, PG may play an essential role in the regulation of nuclear transcription signals.

#### 4.3.1. PG Acts as a Suppressor Gene in HNC

As a strong adhesion and signaling molecule, altered PG has been associated with various diseases, such as skin, heart, and certain types of cancer. In the tissue models of tongue tumorigenesis at different stages constructed in vitro, the immunostaining intensity of PG decreased with the progression of the disease [47]. In OSCC, reduced γ-catenin expression was associated with poor differentiation, lymph node metastasis, and poor survival [80,81,142]. In oropharyngeal SCC, PG immunoreactivity showed that abnormal cytoplasmic localization was negatively correlated with tumor size and was directly associated with poor patient outcomes [143]. In addition, both integrins (ITG) and JUP are located around the cell membrane, and their expression ratio may reflect the tumor stage of SCC. Multivariate logistic regression analysis showed that the expression of ITGA3/JUP was a significant factor affecting lymph node metastasis of tongue squamous cell carcinoma (TSCC). Furthermore, a high ITGB4/JUP level showed a significantly higher mortality rate [144], which was also a significant factor for distant metastasis [145]. These results suggest that ITGA3/JUP and ITGB4/JUP ratios are potentially effective biomarkers for predicting lymph node metastasis and prognosis of HNC.

Many reports have suggested that PG can act as a tumor/metastasis suppressor in HNC. The signal transduction activity of PG can be regulated in combination with various intracellular partners (Figure 3). Firstly, the subcellular distribution of PG has a regulatory effect on the carcinogenic potential of β-catenin. In the cytoplasm, PG can substitute the role of β-catenin in the adhesion complex by interacting with α-catenin, releasing the oncogenic form of β-catenin, whereas, in the nucleus, PG competes with TCF-activated β-catenin [94,100]. Moreover, the expression of Bcl-2 was induced by β-catenin and regulated by PG distribution [100]. Secondly, PG interacts with the tumor suppressor non-metastatic protein 23 (Nm23), in which α-catenin acts as a bridge. Furthermore, PG can display partial tumor-suppressive activity by regulating Nm23 expression and subcellular localization [101]. Thirdly, PG can interact with the transcription factor p53 in the cytoplasm and nucleus, promote the transcriptional activity of p53, and bind to the p53 consensus sequence in the 14-3-3σ promoter to regulate the expression of 14-3-3σ [102]. In addition, the oncogenic chromatin remodeling factor SATB1 has been identified as another target gene of PG and p53, which negatively regulates the expression of SATB1. In support, overexpression of PG inhibited cell proliferation, migration, and invasion [103]. In conclusion, PG is involved in the development of HNC by regulating the expression of multiple genes.

#### 4.3.2. PG Acts as an Oncogene in HNC

Even though extensive studies have claimed that PG is a suppressor in HNC, there was a report showing that PG overexpression was associated with a poor prognosis for OSCC, indicating an independent prognostic factor [79]. It suggests that PG is oncogenic in HNC. 

In OSCC, stable overexpression of JUP can promote cell proliferation, invasion, migration, and inhibit cell apoptosis, but the specific molecular mechanism remains unclear [79]. Consistent with the above, overexpression of PG in PG-deficient TSCC cells (SCC9) leads to uncontrolled growth and inhibition of apoptosis, induction of expression of Bcl-2, and inhibition of caspase three cleavages [99].

#### 4.3.3. The Nuclear Translocation of PG

The localization of PG in desmosome proteins mediates cell-cell adhesion, whereas the cytoplasmic/nuclear form plays a role in signal transduction. PG is shown to exhibit β-catenin-like activity and modulate Wnt/β-catenin signaling. LEF/TCF transcription factors mediate the Wnt signaling in the nucleus by recruiting β-catenin, which plays a vital role in cell proliferation, survival, and migration [146]. Aberrant Wnt/β-catenin/TCF pathways have been implicated in the progression of various diseases, including cancer [147,148]. DSG3 silencing increases PG translocation in the nucleus, where it interacts with TCF/LEF and inhibits transcriptional activity to suppress carcinogenesis in HNC cells [94]. Correspondingly, overexpression of Dsg3 contributes to β-catenin-LEF/TCF interaction and activation [94]. In support, other researchers indicated that PG negatively regulates the Wnt/β-catenin/TCF signaling pathway [149]. In line with this notion, in mouse models of chronic rhinosinusitis (CRS), Dsg3 silencing inhibited inflammation by disrupting the Wnt/β-catenin signaling pathway [150].

### 4.4. Desmoplakin

DSP is a necessary structure connecting desmosome core protein and intermediate filament skeleton and is also the most abundant component in desmosomes. DSP has spherical amino terminus and carboxyl terminus connected by α-helical coiled-coil rod domain. The amino-terminal domain provides binding sites for PG and PKP [151], and the carboxylate terminal contains three plakin repeat domains (A, B, C) and a glycine-serine-arginine-rich domain (GSR) that regulates the binding of DSP to intermediate filaments [152] (Figure 2). Like DSC, DSP produces DSP I and II by selective splicing of RNA. Two subtypes of DSP are widely expressed in many tissues, and tissue-specific mouse knockout studies have shown that Dsp plays a crucial role in the skin and heart [153].

#### 4.4.1. DSP Is Modulated by Signaling Molecules to Regulate Cell Adhesion

Alterations in the expression or function of DSP may affect desmosome assembly and signal transduction of cancer cells, which may promote tumorigenesis. Compared with the control group, the invasion and motility of OSCC cells overexpressing Kallikrein-related peptidase 13 (KLK13) were decreased, accompanied by up-regulation of adhesion molecules PG, PKP4, DSC2, DSG2, and DSP [154]. Additionally, in OSCC cells treated with EGFR inhibitors pKI166 and C225, DSP aggregates to the cell-cell borders and increases in the triton-insoluble cell fraction and contributes to the association of the IF network with cell-cell attachment sites, supporting the idea that inhibition of EGFR enhances desmosome assembly [90]. These reports suggest that signal transduction molecules influence the motility of cancer cells by altering the adhesion function of DSP, but the exact molecular mechanism remains to be determined.

#### 4.4.2. Prognostic and Metastatic Potential of DSP in HNC

Previous studies have shown that DSP protein dysregulation affects tumor behavior in various human cancers, including OSCC. DSP is downregulated in human OSCC (Table 1), and the decrease in DSP staining is associated with loss of differentiation, degree of invasion, and the presence of lymph node metastasis [71,72]. In the dysplasia tissue, the basal cells were moderately stained, the upper differentiated cells were intensely stained, and in the malignant tissue, the whole section was weakly stained [47]. Moreover, the two DP isoforms showed different subcellular distribution patterns, and the immunoreactivity to DPII was detected in patients with abnormal cytoplasmic localization [82]. Analysis of OSCC using global proteomics showed that the RNA of DSP was strongly correlated with encoding proteins. Its reduction was associated with a significantly shorter time to distant metastasis [77]. These findings suggest that DSP may serve as a biomarker to assess prognosis and metastatic risk of HNC.

## 5. Concluding Remarks

Desmosomal components play diversified roles in the development of HNC. The expression and function of desmosome cadherin, armadillo protein, and the desmosome protein subfamily are not consistent in HNC. Although it is unclear what causes this inconsistency, several physiological and pathological factors, molecular modifications, interacting proteins, and subcellular locations seem to determine their role in HNC.

PTM, proteolytic cleavage, and the biogenesis of regulating EVs may be the critical factors for the role of DSG2 protein in HNC. EVs are messengers in the intercellular signaling system and play a key role in tumorigenesis and metastasis by altering TME [155]. EVs have great potential in clinical applications, such as manipulation of tumor genetic pathways [156], tracking the progression of various pathological states as a biomarker [157], and regulating cell function in vivo [158]. Future studies will focus on how DSG2-regulated EV biogenesis can be applied to clinical studies. In addition, studies have confirmed that activation of MMP and ADAM can participate in the shedding of the extracellular domain of DSG2, and hydrolysis of DSG2 interacts with HER2 or HER3 to activate Akt/mTOR and MAPK (mitogen-activated protein kinase) signaling pathways, promoting the proliferation of intestinal epithelial cells (IEC) [159]. However, the role of DSG2-cleaved fragments in cancer and the effect of this hydrolytic expression pattern on HNC progression requires further investigation. Given that DSG2 enhances oncogenesis, PTM may play a role in the ability of DSG2 to promote these properties, but the specific molecular mechanisms need to be clarified.

Overexpression of DSG3 may be conducive to its oncogenic signaling activity, leading to accelerated movement and proliferation of cancer cells. However, many questions remain unanswered. For example, what mechanisms regulate its gene expression in cancer cells? Is the overexpression of DSG3 in cancer involved in post-translational regulation, such as DSG2? Interacting proteins and nuclear translocation may dominate the role of PG in HNC. Several desmosomal components, such as PKP1, PKP3, and DP, appear to be responsible for the gene/protein dysregulation in the development or/and cell-cell adhesion of HNC.

Recently, the non-adhesion and non-junction functions of desmosomal components in signal transduction of HNC deserve attention. In addition to interactions between desmosome protein molecules, identifying new acting partners can affect downstream signaling networks. Currently, there is only limited evidence of specific roles of desmosome proteins in certain features, characterization of new participants, the role of post-translational modifications, and identification of novel signaling pathways that will contribute to a better understanding of the role of desmosome HNC progression. Additionally, HPV is a newly identified causal factor for HNC and other cancers, and its effect on desmosomal components should be examined. In cervical cancer, the high expression of DSG2 was associated with HPV-positive status [160], suggesting that DSG2 may be involved in HPV-induced cervical carcinogenesis. Varga et al. [161] revealed that DSG3, as one of prognostic panel genes, has the ability to differentiate high-risk HPV-positive CIN1 (cervical intraepithelial neoplasia) and cancer cases. E6 and E7 early gene products contribute to the oncogenic potential of high-risk HPV [162]. Eszter et al. [163] found that the expression of DSC1 was significantly downregulated in the presence of HPV 16 E6 and E7 oncoprotein. Mechanism studies have shown that HPV oncoprotein can downregulate the transcriptional activity of the promoter of the DSC1 gene. However, the effect of HPV infection on desmosomal components and its mechanism in HNC have not been reported; this remains to be further studied.

We hope that summarizing and analyzing desmosome molecules can help better understand how these factors are involved in the development and growth of HNC and further provide some helpful hints for making potential factors as therapeutic targets or diagnostic/prognostic markers.

## Figures and Tables

**Table 1:** The expression of desmosome and their prognostic values in HNC.

Desmosomal Component	Relative Level in HNC	Localization in HNC	Prognostic Values *	Clinicopathological Parameters	Reference(s)
DSC1	High	N. A	Poor	Survival and Differentiation	[63]
DSC2	Low	Cytoplasm	Poor	Lymph node metastasis	[65]
DSC3	Low	Cytoplasm(diffuse), Surface layer	Good	Differentiation	[66,67]
DSG1	Low	Cytoplasm, Basal layer	Good	Survival	[15,67,68]
DSG2	Low	Cytoplasm(mainly)	Poor	Distance metastasis	[73]
DSG2	High	Membrane and cytoplasm(mainly)	Poor	Survival	[65,74,75]
DSG3	Low	Cytoplasm	Poor	Lymph node metastasis	[65,66]
DSG3	High	Cytoplasm	Poor	Survival	[26,76]
PKP1	High	N. A	N. A	N. A	[27]
PKP1	Low	Membrane (32%) and cytoplasm (24%)	Good	Survival	[64,77]
PKP2	High	Membrane (38%) and cytoplasm (57%)	Good	Metastasis	[64]
PKP3	Low	Membrane (8%) and cytoplasm (62%)	Good	Survival and stage	[64,78]
PG	High	N. A	Poor	Overall survival	[79]
PG	Low	Basal layer and parabasal layer	Poor	Survival	[67,80,81]
DSP	Low	Membrane and cytoplasm	Poor	Distant metastasis	[82]

* “Poor” refers to the high expression of desmosome molecules that predict a poor prognosis in patients with HNC, while “good” refers to the high expression of desmosome molecules that predict a good prognosis in patients with HNC.

**Table 2:** The function and mechanism of desmosomes in HNC.

Desmosomal Component	Function	Regulated Signaling Pathway	Reference(s)
DSC1	Low expression of DSC1 decreases proliferation and invasion.	When suppressing DSC1, the expression levels of β-catenin, c-myc and cyclin D1 proteins were decreased.	[63]
DSC3	The high expression of DSC3 increased cell adhesion.	Membrane localization of DSC3 was significantly enhanced in TP53 mutated tumors.	[84]
DSG1	Cleavage of DSG1 can promote loss of junctional integrity.	Overexpression of KLK-5 expression induces processing of DSG1.	[85]
	DSG1 suppresses invadopodia formation and matrix degradation.	DSG1 interaction with Erbin downregulates invadopodia signaling by dampening EGFR/Erk activation.	[86]
DSG2	Knockdown of DSG2 promotes cell migration and invasion in ATC cell lines.	Depletion of DSG2 activates the HGF signaling pathway (c-Met/Src/Rac1).	[73]
	Secreted DSG2 enhances tumor development.	DSG2 promotes EV release, down-regulation of miR-146a increases in IL-8 expression.	[87]
	DSG2 enhances the mitogenicity of EVs to enhance fibroblast cell growth.	DSG2-EVs activated Erk1/2 and Akt signaling.	[88]
	Reduced DSG2 levels correlated with the diminished strength of cell-cell adhesion.	DSG2 Protein is diminished by proteasome inhibition (Bortezomib).	[89]
	PKI166 treatment results in specific accumulation of DSG2 to cell-cell borders.	Inhibition of EGFR down-regulates MMP-dependent breakdown of DSG2.	[90]
	DSG2 aggregates at the cell-cell borders and enhances intercellular adhesion.	Inhibition of EGFR and MMP interferes with the accumulation of DSG2 in internalized cytoplasmic pools.	[91]
DSG3	A high calcium-associated DSG3 induction enhanced cetuximab efficacy.	Cetuximab treatment increased DSG3 expression.	[92]
	DSG3 promotes cell migration and invasion.	DSG3 regulates the activity of c-Jun/AP-1 as well as PKC-mediated phosphorylation of Ezrin-Thr567.	[93]
	DSG3 silencing suppressed the growth of xenografted tumors.	Knockdown of DSG3 increased the interaction of plakoglobin with TCF and suppressed the TCF/LEF transcriptional activity.	[94]
	Dsg3/γ-catenin involved in growth regulation malignant oral epithelial cells.	Dsg3/γ-catenin showed a mild to severe decrease of membranous and gain of cytoplasmic expressionforming characteristic perinuclear aggregations in OSSCs.	[95]
PKP1	The loss of PKP1 expression in OSCC cells results in increased cell motility and invasion.	Decreased PKP1 levels are accompanied by DSP redistribution from cell borders to a diffuse cytoplasmic localization.	[96]
	Loss of PKP1 altered cell-cell adhesion.	Expression of Slug induced EMT characterized by a cadherin switch and loss of desmosomal adhesion.	[97]
PKP3	Low expression of PKP3 is associated with NPC progress.	DNP decreased PKP3 was verified to be through upregulating miR-149.	[69,98]
PG	The high expression of PG promoted the growth and inhibited the apoptosis of SCC9 cells.	Overexpression of PG in SCC9 increased expression of Bcl-2 and inhibition of caspase 3 cleavage.	[99]
	The expression of Bcl-2 was induced by β-catenin and regulated by PG.	The presence of PG in the nucleus decreases the level of nuclear β-catenin.	[100]
	PG can display partial tumor suppressive activity.	PG regulates the expression and subcellular localization of Nm23.	[101]
	PG promoted the transcriptional activity of p53 and induced 14-3-3σ expression.	PG binds to the p53 consensus sequence in the 14-3-3σ promoter.	[102]
	PG reduces cell growth, proliferation, invasion and migration.	PG inhibits the expression of SATB1.	[103]
DSP	DSP were recruited to cell-cell borders.	EGFR blockade promotes desmosome assembly.	[90]

## Abbreviations

ADAM, a disintegrin and metalloprotease; AP-1, activator protein 1; ATC, anaplastic thyroid cancer; BMI, body mass index; CAV1, caveolin-1; CIN1, cervical intraepithelial neoplasia; CRS, chronic rhinosinusitis; DMBA, 9.10-dimethyl-1.2-benzoanthracene; DMF, N,N-dimethylformamide; DNP, N,N′-dinitrosopiperazine; DSC, desmocollin; DSG, desmoglein; DSP, desmoplakin; ECs, extracellular domains; EGFR, epidermal growth factor receptor; EMT, epithelial mesenchymal transformation; Erbin, ErbB2 Interacting Protein; EVs, extracellular vesicles; GSR, glycine-serine-arginine rich domain; HB-EGF, heparin-binding epidermal growth factor; HGFR, hepatocyte growth factor receptor; HNC, Head and neck cancer; HNSCC, head and neck squamous cell carcinoma; HPV, human papillomavirus; IA, intracellular anchor; ICS, cadherin-typical sequence; IEC, intestinal epithelial cells; IF, intermediate filaments; IL-8, Interleukin 8; ITG, integrins; KLK5, kallikrein 5; KLK13, Kallikrein-related peptidase 13; MAPK, mitogen-activated protein kinase; MMP, matrix metalloprotease; Nm23, non-metastatic protein 23; NPC, nasopharyngeal carcinoma; OL, oral leukoplakia; OSCC, oral squamous cell carcinoma; PERP, p53 apoptosis effecter related to the PMP-22; PG, plakoglobin, PKC, protein kinase C; PKP, plakophilins; PL, proline-rich linker; PTM, post-translational modification; RUD, repeat unit domain; SCC, squamous cell carcinomas; TM, transmembrane domain; TSCC, tongue squamous cell carcinoma; UVB, ultraviolet B
